# Transcatheter Pulmonary Valve Implantation in Congenital Heart Diseases: Current Advances and Future Prospectives

**DOI:** 10.3390/children12050547

**Published:** 2025-04-24

**Authors:** Mario Giordano, Raffaella Marzullo, Gianpiero Gaio, Maurizio Cappelli Bigazzi, Giovanni Domenico Ciriello, Maria Teresa Palladino, Berardo Sarubbi, Maria Giovanna Russo

**Affiliations:** 1 Pediatric Cardiology Unit, AORN “Ospedali dei Colli”, Monaldi Hospital, University of Campania “Luigi Vanvitelli”, 80131 Naples, Italy; raffaella.marzullo@ospedalideicolli.it (R.M.); gianpierogaio@hotmail.com (G.G.); maurizio.cappelli@ospedalideicolli.it (M.C.B.); mariateresa.palladino@ospedalideicolli.it (M.T.P.); mariagiovanna.russo@ospedalideicolli.it (M.G.R.); 2 Adult Congenital Heart Disease Unit, AORN “Ospedali dei Colli”, Monaldi Hospital, Via Leonardo Bianchi n.1, 80131 Naples, Italy; gdciriello@gmail.com (G.D.C.); berardo.sarubbi@ospedalideicolli.it (B.S.)

**Keywords:** interventional cardiology, grow-up congenital heart disease, pulmonary obstruction, pulmonary regurgitation, transcatheter valve replacement

## Abstract

Pulmonary disfunction is frequent in repaired congenital heart diseases. Both pulmonary regurgitation and pulmonary stenosis are possible complications over time. In the past, the surgical approach was the only feasible management but exposed the patient to a redo-surgery with its consequent risks. Nowadays, the development of novel devices and techniques has made possible a transcatheter pulmonary valve implantation. The Melody Transcatheter Pulmonary Valve (TPV) (Medtronic Inc., Minneapolis, MN, USA) and the Edwards Sapien XT and S3 Transcatheter Heart Valve (Edwards Lifesciences LLC, Irvine, CA, USA) are balloon-expandable valvular bioprostheses approved for pulmonary position. Venus P-Valve (Venus Medtech, Shanghai, China) and Harmony TPV (Medtronic Inc., Minneapolis, MN, USA) are self-expandable pulmonary valves. Alterra Adaptive Prestent (Edwards Lifesciences LLC, Irvine, CA, USA) is an hourglass self-expandable stent that reduces the size of large right ventricular outflow tracts, creating a suitable landing zone to implant an Edwards Sapien S3 THV 29 mm. Novel stents and percutaneous valves are being planned and experimented with to widen the field of transcatheter approach. The aim of this review is to describe both the current approaches, strategies, and techniques as well as the future perspective to deal with the patients with significant pulmonary stenosis and/or regurgitation.

## 1. Introduction

Surgical reconstruction of the connection between the right ventricle and the pulmonary artery is required in various congenital heart diseases (CHDs): tetralogy of Fallot, pulmonary atresia with ventricular septal defect, truncus arteriosus, transposition of great vessels with ventricular septal defect and pulmonary stenosis/atresia, and various forms of double outlet right ventricle. A transannular patch allows an optimal correction of right-sided obstruction. However, the implantation of a conduit is sometimes necessary to restore a connection between the right ventricle (RV) and the pulmonary artery (PA). Furthermore, patients affected by valvular aortic stenosis may undergo a Ross operation (replacement of the aortic valve with a pulmonary allograft), needing a conduit to rebuild the RV-PA connection. These patients may develop significant surgical sequels over time. A hemodynamically significant severe regurgitation is a typical consequence of trans-annular patch repair, whereas a conduit degeneration may determine a significant obstruction [1]. However, even a dysfunctional conduit may develop a severe regurgitation. Numerous causes of conduit stenosis have been described: valve degeneration, conduit-patient mismatch, proximal/distal anastomosis stenosis, conduit calcification, external compression, isolated ostial stenosis of the PA, and endocarditis [1].

In these settings, a pulmonary valve replacement is necessary to avoid a poor patient’s prognosis. In the past, the surgical pulmonary valve replacement (SPVR) was the only chance to deal with a pulmonary dysfunction; whereas, currently, the transcatheter pulmonary valve replacement (TPVR) is the first choice [1].

Melody Transcatheter Pulmonary Valve (TPV) (Medtronic Inc., Minneapolis, MN, USA), Edwards Sapien (XT and S3) Transcatheter Heart Valve (THV) (Edwards Lifesciences LLC, Irvine, CA, USA), Venus P-Valve (Venus Medtech, Shanghai, China), Harmony TPV (Medtronic Inc., Minneapolis, MN, USA) and Alterra Adaptive Prestent (Edwards Lifesciences LLC, Irvine, CA, USA) are available and approved by both the Food and Drug Association (FDA) and the European Community (CE mark) for percutaneous pulmonary valve replacement.

The objective of this review is to highlight the features, the technical advances, and the outcomes of TPVR and to explore the future prospectives in this field.

## 2. Indications for Pulmonary Valve Replacement

The last 2020 ESC guidelines about adults with congenital heart diseases (ACHDs) [2] suggest that the pulmonary valve replacement:

- is recommended (class I, level C) in symptomatic patients with severe pulmonary regurgitation (CMR regurgitant fraction > 30–40%) and/or at least moderate right ventricle outflow tract (RVOT) obstruction (echo Vmax > 3 m/s);
- should be considered (class IIa, level C) in asymptomatic patients with severe pulmonary regurgitation and/or RVOT obstruction when a decrease in objective exercise capacity and/or a significant right ventricle dilatation (RV end-systolic volume index ≥ 80 mL/m^2^, RV end-diastolic volume index ≥ 160 mL/m^2^) and/or a tricuspid valve regurgitation progression (at least moderate), and/or a progressive RV systolic dysfunction, and/or a very high right ventricle systolic pressure (>80 mmHg) are detected.

According to the American College of Cardiology and the American Heart Association [3], pulmonary valve replacement is indicated in the following cases:

- symptomatic patients with moderate or severe regurgitation (class I)
- asymptomatic patient with moderate or severe pulmonary regurgitation and two or more of these elements: mild or moderate right or left ventricle systolic dysfunction; severe RV dilatation (RV end-diastolic volume index ≥ 160 mL/m^2^ or RV end-systolic volume index ≥ 80 mL/m^2^ or RV end-diastolic volume—left ventricle end-diastolic volume ratio ≥ 2); RV systolic pressure ≥ 2/3 of systemic arterial pressure due to RVOT obstruction; progressive reduction in objective exercise tolerance (class IIa)
- asymptomatic patients with moderate or severe pulmonary regurgitation and sustained tachyarrhythmias (class IIb)
- Asymptomatic patients with moderate or severe pulmonary regurgitation and residual lesions requiring surgical treatment (class IIb).

Therefore, both European and American ACHD guidelines agree that the TPVR should be preferred to surgery, and the optimal timing is challenging, as most studies have focused on preoperative RV volumes that would result in normalization of post-operative volumes, but these cutoffs might not be correlated with a clinical improvement of the patients [4,5,6,7].

In literature, several authors explored the outcomes of a proactive versus a conservative approach to pulmonary valve replacement (PVR). The proactive and conservative approach criteria to PVR are summarized in Table 1 [8].

Recently, a propensity score-adjusted analysis of the International Multicenter TOF Registry (INDICATOR) [9] identified favorable outcomes after PVR for all-cause mortality, cardiac arrest, or sustained ventricular tachycardia (VT) (defined as VT lasting > 30 s or requiring cardioversion) in patients fulfilling proactive criteria.

The “proactive criteria” indications consider “forgotten parameters” by the guidelines (e.g., QRS width, right and left ventricle ejection fraction). However, additional factors, such as tricuspid regurgitation grade, myocardial fibrosis, RV pressure, right atrial size, aerobic capacity, and genetic influences, may help to refine the optimal timing of PVR [9].

The analysis of the Canadian Outcomes Registry Late After Tetralogy of Fallot Repair (CORRELATE) [10] demonstrated right ventricle fibrosis is associated with cardiac reverse remodeling (increased RV end-systolic volume, RV mass, and right atrial dimensions, specifically) following PVR. Therefore, RV late gadolinium enhancement (LGE) is the most important independent predictor of mortality and sudden cardiac arrest in patients with repaired TOF, suggesting a key role of RV fibrosis in the prognosis of these patients [11]. PVR reverses these fibrotic changes, reducing the risk of sudden death [12].

By a multivariable Cox model analysis of the INDICATOR, Mayourian et al. [13] identified older age, obesity (BMI ≥ 30 kg/m^2^), type of TOF repair, higher RV end-systolic volume index, and lower biventricular global function index as independent predictors of death in repaired TOF. Using these variables, a mortality risk score (available online, https://github.com/rTOF-INDICATOR/Mortality-Risk-Score, accessed on 21 April 2025) was built. The score identified a low-risk (score ≤ 4; 15-year survival of 95%) and a high-risk (score > 4; 15-year survival of 74%, *p* < 0.001) group.

Recently, Geva et al. [14] proposed a novel algorithm about the management and the indication for the PVR in repaired TOF, considering the INDICATOR score in the decision algorithm.

In the future, the development of specific trials on the topic will certainly help to identify the optimal timing of pulmonary valve replacement and maybe to use different indications for surgical or percutaneous replacement.

## 3. Melody Transcatheter Pulmonary Valve

**Figure 1:**
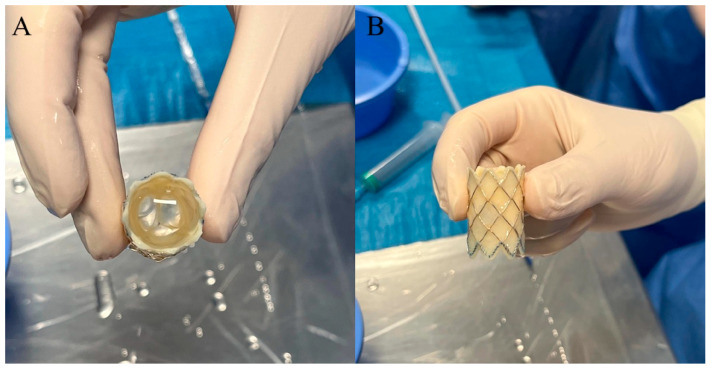
Frontal (**A**) and lateral (**B**) view of Melody TPV.

**Figure 2:**
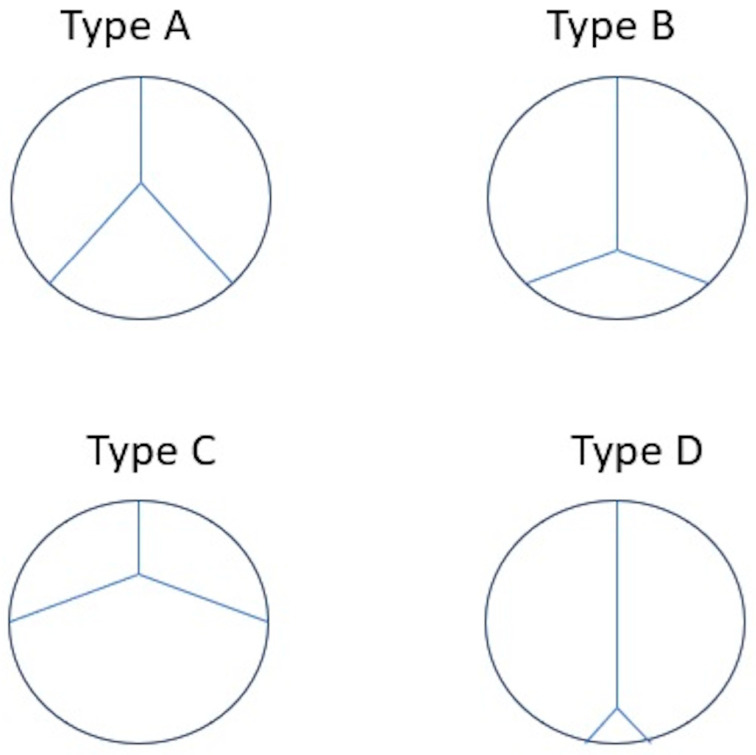
Leaflet morphology types of Melody TPV: symmetric tri-leaflets (type A), asymmetric tri-leaflets with a single small leaflet (type B), asymmetric tri-leaflets with a single large leaflet (type C), and rudimentary leaflet with near bicuspid appearance (type D).

Melody TPV [15] is a balloon-expandable biologic valvular prosthesis made of a valved bovine jugular vein sutured within a closed-cell Cheatham Platinum (CP) stent (NuMED Inc., Hopkinton, NY, USA) [Figure 1]. Various leaflet morphology types of Melody TPV have been identified: symmetric tri-leaflets (type A, 47%); asymmetric tri-leaflets with a single small leaflet (type B, 32%); asymmetric tri-leaflets with a single large leaflet (type C, 16%); and rudimentary leaflets with a near bicuspid appearance (type D, 5%) [Figure 2]. However, leaflet morphology does not influence the outcomes [16].

The valvular prosthesis is crimped onto a specific delivery system characterized by a balloon-in-balloon catheter: the Ensemble Transcatheter Delivery System (22 Fr). This latter one is available in three different sizes representing the diameter of the outer balloon: 18 mm, 20 mm, and 22 mm. This delivery system allows an accurate deployment of the valve: the inner balloon is inflated first, the valvular deployment is checked, and then the outer balloon is inflated to release the Melody TPV.

Currently, two different sizes of Melody TPV are available: 16 mm and 18 mm, which are expandable up to 20 mm and 22 mm, respectively [Table 2]. However, the valvular bioprosthesis is continent up to 24 mm [17].

According to Medtronic Instructions for Use (IFU), Melody TPV should be used to deal with significant stenosis and/or regurgitation of both the RVOT conduit and the bioprosthetic pulmonary valve. However, the off-label use of the Melody TPV into native/patched has been adopted in many circumstances [18]. In addition, Melody TPV has also been implanted safely in large outflow tracts [19,20], in small conduits [21,22], in small children [22,23,24], and even in other locations (such as tricuspid or mitral positions) [25,26].

The most contraindications to Melody TPV are active endocarditis, significant obstruction of the central veins, and a venous system unable to accept a 22 Fr size delivery system.

In most cases, it is required to interrogate the landing zone before the Melody TPV implantation. Serial balloon dilatation is performed into the RVOT conduit up to reach the potential final diameter of Melody TPV, and an injection in the aortic root is performed at the same time, excluding coronary compression. The aortography is usually sufficient to achieve an adequate view of the coronary artery and of eventual compression. However, if any doubt persists, a selective coronary angiography may be performed to be sure of the absence of coronary compression. Compression of the aorta has also been observed and, in its most severe form, can be a contraindication for transcatheter intervention [27].

Following coronary assessment, a covered CP stent is usually placed to avoid mid-term stent fracture, to minimize the risk of tear of the conduit, and other RVOT reintervention [28,29].

**Figure 3:**
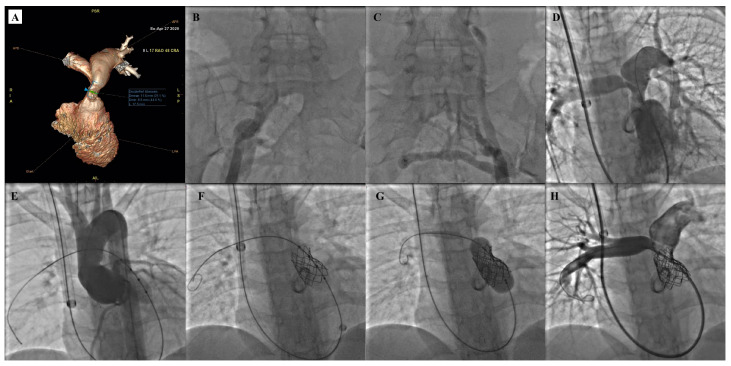
Procedural steps of Melody TPV implantation. Angio-CT scan detection of a significant stenosis of an RV-PA conduit (**A**). Bilateral thrombosis of femoral veins (**B**,**C**) forced them to adopt the right jugular vein as vascular access. Pulmonary artery injection highlights a significant stenosis of the RV-PA conduit (**D**). RVOT balloon interrogation identifies the lack of coronary artery compression (**E**). RV-PA conduit pre-stenting (**F**) is followed by Melody TPV implantation (**G**). Pulmonary artery injection highlights a well-deployed continent Melody TPV (**H**).

The procedural steps of Melody TPV implantation are illustrated in Figure 3.

A simultaneous pre-stenting and Melody TPV implantation may be adopted to reduce global procedural and fluoroscopic times and radiation exposure, since it is not associated with higher risks of adverse events [30].

During a decade of follow-up, the Melody TPV showed favorable long-term outcomes with high durability and low rates of reintervention. In the largest retrospective study cohort (supported by the British Heart Foundation), most patients were free of surgery (80%) or any reoperation (55%) at 10 years of follow-up with a significant improvement in functional status [31]. In the cases of Melody TPV stenosis due to stent recoil, a percutaneous balloon dilatation with high-pressure balloons may be effective to restore an adequate valvular function, whereas the patients with stent fracture and/or Melody TPV insufficiency require a transcatheter valve-in-valve implantation [32]. In stenosis due to patient-prosthesis missmatch, surgical replacement is the only adoptable strategy.

The infective endocarditis remains the most important concern. The incidence rate of Melody TPV endocarditis varies considerably among the studies (from 1% to 2.4% per patient-year [33,34]) and is higher than the pulmonary homograft one [35]. Immunocompromised status was the most significant predictor of infective endocarditis development post-Melody TPV [34]. Recent reports suggest that a long-lasting acetylsalicylic acid therapy, an implantation age > 12 years, and a residual peak-to-peak gradient < 15 mmHg are associated with a lower incidence of infective endocarditis [34,35,36,37].

## 4. Edwards Sapien Transcatheter Heart Valve

The Edwards Sapien THV is a balloon-expandable valvular bioprosthesis, characterized by three bovine pericardial cusps mounted within a stainless-steel stent.

Two kinds of Edwards Sapien THV are commercialized: Edwards Sapien XT and Edwards Sapien 3 THV (and Edwards Sapien 3 Ultra THV). The former has a cobalt-chromium frame with a large cell design and no commissural posts, which gives high radial strength, whereas the latter has a cobalt-chromium frame with a smaller cell design and has three commissural posts. The new generation valves also have an outer polyethylene terephthalate cuff, reducing the risk of paravalvular leak. Finally, its stent has different geometries in the proximal and distal portions so that it shortens even more in its proximal border.

All Edwards Sapien valvular bioprostheses undergo a glutaraldehyde fixation process to prevent calcifications.

Three sizes of Sapien S3 THV are currently available: 20, 23, 26, and 29 mm, and they are delivered by the Edward Commander system [Table 3].

The Edwards Sapien THV has gained popularity due to its greater versatility in terms of sizes exceeding the maximal diameter of the Melody TPV. This advantage has contributed to expanding its use in native, patched, and larger RVOT (up to 29 mm of diameter) [38,39,40].

In the past, pre-stenting was recommended by the technical schedule before bioprosthesis implantation. However, recently, direct valvular implantation is the most adopted technique when an adequate landing zone is detected and there is a lack of significant stenosis [39,40,41].

The choice of bioprosthesis size is related to the “balloon interrogation/occlusion test”. A sizing balloon is inflated into the target zone to achieve a strong waist. Usually, high-pressure balloons are used to stretch conduits, while semi-compliant balloons are used for native outflow tracts. The waist diameter addresses the valvular size: Edwards Sapien THV 20 mm for waists of 18–20 mm, Edwards Sapien THV 23 mm for waists of 20–22 mm, Edwards Sapien THV 26 mm for waists of 23–25 mm, and Edwards Sapien THV 29 mm for waists of 26–28 mm. The overexpansion of the valve with an additional 5 mL volume is used to reach the diameter of 30 mm and to reduce the risk of embolization or paravalvular leak [42]. During the inflation of the sizing balloon, a right ventricular injection is performed to ensure a pulmonary occlusion. Contemporarily, an aortography is performed to exclude signs of aortic root and/or coronary compression that are contraindications to deploying the transcatheter valvular bioprosthesis.

The delivery system (22 or 24 Fr) consists of a long delivery sheath with a dilator and a tapered steerable tip. The handle has a knob to steer the tip of the catheter. A balloon catheter is used to release the valve.

To facilitate the delivery of the Edwards Sapien THV during transcatheter pulmonary valve replacement and reduce the risk of tricuspid valve damage, it has been proposed to use 65 cm large-caliber Dryseal sheaths (W. L. Gore & Associates, Flagstaff, AZ, USA) [43]. The 65 cm Dryseal sheath is a hydrophilic, flexible, kink-resistant sheath (available from 12 Fr to 26 Fr) that is advanced up to the main pulmonary trunk. The inner dilator is removed, and the Edwards Sapien THV (crimped onto its own delivery system outside the patient) is advanced into the Dryseal sheath up to the landing zone and then released. This approach facilitates the procedure, decreasing the risk of adverse events, procedural time, and radiation dose. Furthermore, the Dryseal sheath allows for contrast injection during valve implantation to check the deployment accurately.

**Figure 4:**
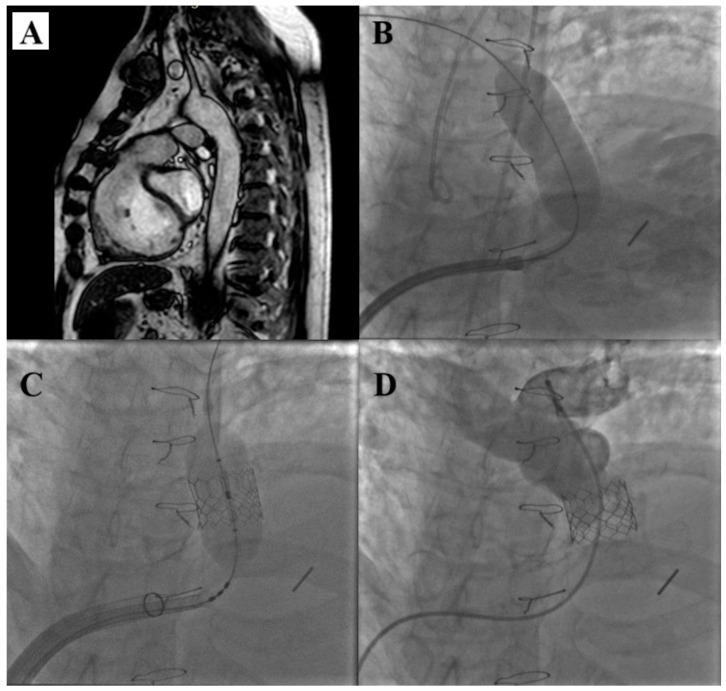
Direct Edwards Sapien 3 implantation. Cardiac MRI highlights a severe pulmonary regurgitation with a significant right ventricle dilatation (**A**). RVOT balloon interrogation with an occlusive semi-compliant 28 mm balloon (**B**). A direct implantation of an Edwards Sapien 3 29 mm (**C**). Pulmonary artery injection demonstrates a well-deployed continent Edwards Sapien 3 (**D**).

The steps of Edwards Sapien THV implantation are illustrated in Figure 4.

Several multicenter studies supported the feasibility and the mid-term effectiveness of the Edwards Sapien S3 THV, demonstrating the clinical benefits of the procedure, the low incidence of infective endocarditis, and the structural valve integrity over time [40,41,44].

Recent data from the international registry of SAPIEN 3 THV (sponsored by Edwards Lifesciences LLC), including 840 consecutive patients (half of whom had native or patched RVOTs), reports favorable long-term outcomes with low rates of further intervention with a cumulative valve replacement and infective endocarditis risk of 0.7% and 0.5% per patient-year, respectively [45].

## 5. Venus P-Valve

The Venus P-Valve is a self-expandable valvular biological prosthesis characterized by a porcine pericardial tissue valve within a self-expandable Nitinol support frame. The prosthetic valve has an hourglass shape with a narrower mid-tract with the valvular cusps and two wider outer sections (proximal and distal ends). A skirt on the proximal tract has the role of minimizing the risk of paravalvular leak, whereas the distal tract is not covered to minimize the risk of pulmonary branch exclusion. Radiopaque markers border the mid-tract of the prosthesis. Nowadays, there are five models of the Venus P-Valve, and every model is available in two different lengths (25 and 30 mm) [Table 4]. The delivery catheter system is characterized by a distal tip where the bioprosthesis is crimped and a proximal handle to manage it. The handle includes a macro slide grip to open and close the capsule and a micro adjustment knob to facilitate precise bioprosthesis placement. The micro knob is turned counterclockwise to load the bioprosthesis and clockwise to deploy the bioprosthesis.

Contrary to the balloon-expandable valvular bioprosthesis, a preoperative angio-CT and/or cardiac MRI is mandatory. They allow the evaluation of the systo-diastolic excursion and dimensions of the RVOT and main PA by predicting the valvular deployment and the procedural effectiveness [46]. A gated angio-CT and/or cardiac MRI are crucial to identify the RVOT-MPA morphology. Tubular or multiple waist shapes are favourable to implant the Venus P-Valve, whereas a pyramidal or conical shape is unlikely to be suitable for the Venus P-Valve [47]. Nowadays, holography-guided procedural planning [48] and virtual reality [49] may be useful in the management of complex scenarios.

In the catheterization laboratory, an RVOT interrogation with a compliant balloon is useful to profile the shape and valvular landing zone. A semi-compliant balloon interrogation may be performed to predict the risk of aortic root or coronary compression. With the self-expandable valves, this complication is rarer than with the balloon-expandable ones. The middle section diameter of the Venus P-Valve chosen should be 2–4 mm over the size of the main pulmonary artery waist (measured by a compliant balloon interrogation), whereas the expanded full length of the middle section should match the entire main pulmonary artery. By using a large Dryseal sheath (24 or 26 Fr), the Venus P-Valve is released progressively from a pulmonary branch (usually the left one), even if after the final deployment the valvular bioprosthetic will be within the main PA and RVOT.

**Figure 5:**
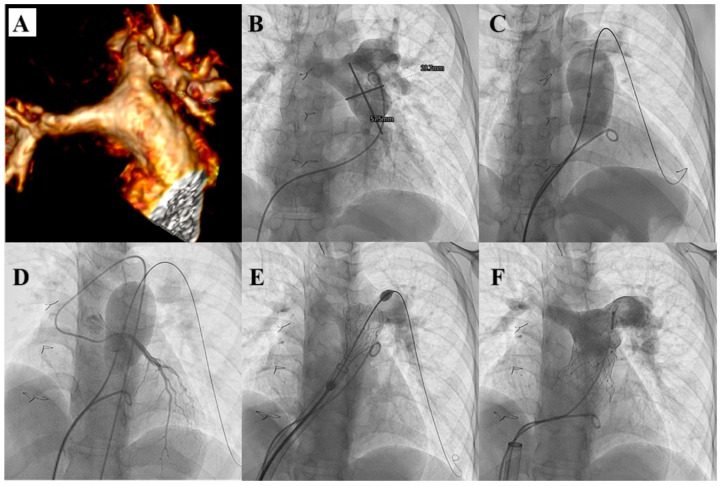
Venus P-Valve implantation. Three-dimensional angio CT scan of a large native RVOT in a repaired tetralogy of Fallot with significant pulmonary regurgitation (**A**). Pulmonary angiography detects the effective diameter and length of the native RVOT (**B**). RVOT balloon interrogation with a compliant balloon demonstrates a 32 mm waist as a landing zone (**C**). RVOT balloon interrogation with a semi-compliant balloon excludes coronary artery compression (**D**). Venus P-Valve implantation (**E**) and pulmonary angiography demonstrating a continent pulmonary valve (**F**).

The procedural steps of Venus P-Valve implantation are illustrated in Figure 5.

There is no agreement about the post-procedural anti-platelet (single or double) or anti-coagulation therapy. A CT scan monitoring during follow-up is necessary to detect potential hypo-attenuated leaflet thickening (HALT) and/or reduced leaflet motion (RELM). The concomitant presence of HALT and RELM identifies a hypoattenuation affecting motion (HAM) that may be indicative of leaflet micro-thrombosis [50]. In this subset, an anticoagulation therapy may be necessary to restore a correct leaflet motion and to avoid the risk of infective endocarditis.

At mid-term follow-up, the Venus P-Valve was associated with an improvement of the patient’s symptoms and a favourable remodelling of the right ventricle [51,52,53].

## 6. Harmony Transcatheter Pulmonary Valve

The Harmony TPV (Medtronic Inc., Minneapolis, MN, USA) is a self-expandable valved covered stent designed to deal with the large native outflow tracts. It consists of a porcine pericardial tissue valve supported by a self-expanding covered stent with a nitinol framework. Two sizes are available: 22 mm and 25 mm. Harmony TPV 22 mm has a 22 mm inner middle valve diameter, a 32 mm distal-end diameter, a 41 mm proximal-end diameter, and a 55 mm length of middle tract. Harmony TPV 25 mm has: a 25 mm inner middle valve diameter, a 43 mm distal-end diameter, a 54 mm proximal-end diameter, and a 51 mm length of middle tract [Table 5]. The Ensemble delivery system (Medtronic Inc., Minneapolis, MN, USA) (25 Fr) has a nosecone that articulates with the capsule but can also be extended beyond the capsule. A pre-operative cardiac magnetic resonance (CMR) or ECG-gated CT scan evaluation is necessary to screen the patients. The preoperative imaging allows us to study the systolic-diastolic excursion of the anatomical system composed of the RVOT and pulmonary artery at multiple levels (outflow tract, sub-valvular tract, mid-valve portion, supra-valvular tract, mid-trunk, and pre-bifurcation portion), achieving a perimeter plot both in systolic and in diastolic phase. This perimeter plot is overlying the Harmony TPV profile. The patient may be treated with this self-expanding valve if all these criteria are satisfied [54]:

- at the distal (outflow) segment, Harmony TPV interference should be ≥15%.
- at the proximal (inflow) segment, Harmony TPV interference should be ≥17%.
- at the midsection, the Harmony TPV should be ideally uncompressed (0% of interference).

In the catheterization laboratory, the Harmony TPV implantation is similar to the Venus P-Valve. A pre-implantation balloon interrogation of the RVOT is helpful to profile the landing zones and to predict an eventual coronary or aortic root compression. A 26 Fr sheath is useful to facilitate the advancement of the delivery system.

The clinical trials (sponsored by Medtronic Inc.) demonstrated that Harmony TPV is able to restore an optimal pulmonary continent [55,56,57]. At 1-year follow-up data, no mortalities, sustained VT, or endocarditis were detected [55,56,57].

## 7. Alterra Adaptive Prestent

The Alterra Adaptive Prestent (Edwards Lifesciences LLC, Irvine, CA, USA) is an internal framework that reduces the size of large right ventricular outflow tracts, creating a suitable landing zone for the implantation of an Edwards Sapien S3 THV 29 mm in the setting of treatment of severe pulmonary regurgitation.

The Alterra Adaptive Prestent is a self-expanding nitinol stent covered with a PET membrane. The device has a symmetrical hourglass shape with the inflow and outflow diameters equal to 40 mm, and the central section measures 27 mm to support the landing zone for an Edwards Sapien S3 THV 29 mm. The distal outflow section has open cells to facilitate the blood flow in case of the stent extension across the origin of a branch pulmonary artery [58]. Currently, suitable anatomy is deemed to have a landing zone diameter between 27 and 38 mm with a minimum length of 35 mm.

The device is loaded in a dedicated delivery system that can be inserted through a 22 Fr long sheath. The delivery system consists of a handle, a retractable outer shaft, and an inner delivery shaft. It allows the operator to recapture two times the pre-stent using a one-handed rotational knob. Once the Alterra device is deployed, an Edwards Sapien S3 THV should be deployed to achieve the pulmonary valve implantation.

The Multicenter Pivotal Study of the Alterra Adaptive Prestent [59] (sponsored by Edwards Lifesciences LLC) demonstrated that the Alterra prestent in combination with the Edwards Sapien S3 THV has excellent outcomes at 2 years follow-up. No reports of deaths, device embolization, coronary artery compression, or endocarditis were described during the follow-up. Thirty-four percent of the patients experienced early post-procedural arrhythmias; most of the early arrhythmias were non-sustained ventricular tachycardia events that resolved with medication. No arrhythmias were noted after 30 days out to 1 year. Interestingly, some patients (17%) reported new or worsening tricuspid regurgitation, which is most likely secondary to tricuspid valve injury, although none of them required intervention. The newly covered pulmonic delivery system should reduce the potential of tricuspid valve injury during these procedures. Finally, improvement in quality of life was detected through the 2-year follow-up.

## 8. Adverse Events of Trans-Catheter Pulmonary Valve Replacement

Adverse events related to TPVR may be both acute and during follow-up. Acute complications are rare (less than 2%), though often life-threatening. The most acute adverse events are coronary compression, aortic root distortion, RVOT injuries and/or conduit rupture, pulmonary edema and significant tricuspid valve regurgitation, stent or bioprosthesis embolization, and interferences.

**Figure 6:**
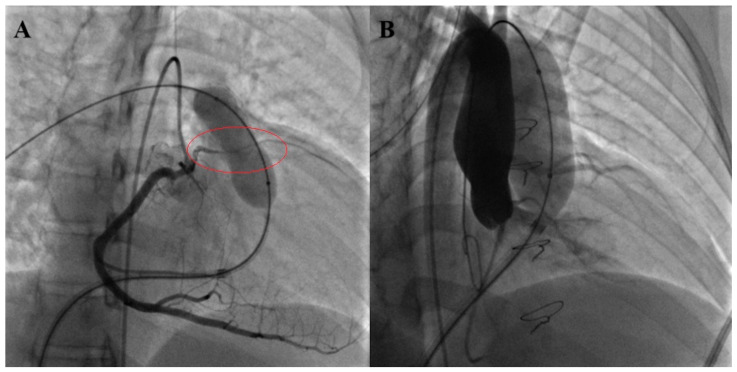
RV-PA conduit balloon interrogation with significant compression of anterior descending artery (red circle) arising by the right coronary artery (**A**). Native RVOT balloon interrogation with a severe aortic root compression (**B**).

Coronary compression and aortic root distortion are very feared complications. Luckily, they may be prevented by an adequate balloon interrogation test before releasing the stent and/or the valvular prosthesis. An aortic root angiogram should be performed to check both signs of aortic root distortion (e.g., new onset aortic regurgitation) as well as compression to the proximal tract of the coronary artery [Figure 6]. A complementary selective coronary angiography may be executed if the aortic root angiogram is not conclusive. The contemporary monitoring of EKG may highlight electrical signs of coronary compression (e.g., ventricular repolarization anomalies). Signs of coronary or aortic root compression impose stopping the percutaneous procedure [Figure 6]. The incidence of coronary compression during the balloon occlusion test is around 6% [60].

RVOT injury and/or conduit rupture are often fatal complications. They may arise both during balloon occlusion tests and during stent/bioprosthesis implantation. An immediate dealing with the complication is the only change to survey for the patient. In a hybrid catheterization laboratory, a rescue switch from a percutaneous procedure to surgical management allows for the lesion to be repaired rapidly. However, in the other cases, the implantation of a covered stent is the only feasible treatment. It is always recommended to have available either a pre-mounted covered stent of adequate diameter or the chance to implant a peripherical extracorporeal membrane oxygenation (ECMO) before the balloon occlusion test or valve implantation. Conduit rupture may occur both with high-pressure balloons and with low-pressure ones. Heavy calcification and homograft conduit were significant risk factors of conduit rupture [61].

Stent and/or bioprosthesis embolization is possible with both the balloon-expandable and the self-expandable valves. Both a proximal embolization towards the RV and a distal migration towards the pulmonary arteries are potential complications. In these circumstances, a balloon-assisted technique may be useful to try to redeploy the valvular bioprosthesis. However, it is often required a surgical management of these adverse events.

Pulmonary oedema is a rare complication due to the sudden increase in pulmonary output in patients with higher left-sided heart filling pressures. In patients with a high risk of developing pulmonary oedema, a preconditioning therapy with diuretics is a helpful strategy. Continuous Positive Airway Pressure (CPAP) is an effective treatment in the cases of severe acute pulmonary oedema [62].

Tricuspid regurgitation may arise immediately after or during a transcatheter pulmonary valve implantation. Direct damage to the tricuspid valve and/or to the subvalvular apparatus (e.g., rope rupture) may determine the onset of acute right heart failure, usually managed with pharmacological therapy (diuretics). The large sheaths that moved inside the right ventricle might be the “weapons” responsible for valvular and subvalvular damage [63,64].

During follow-up, various minor and major complications may arise. The most common complication observed is stent fracture (incidence 21.1%). Three types of stent fracture are described: no loss of stent integrity (type I); loss of integrity with restenosis (type II); and separation of fragments with their embolization (type III). The latter two usually require a new surgical or percutaneous intervention, unlike the first one. Significant risk factors of stent fracture are an implantation into a “native” RVOT, the absence of calcification along the RVOT, and the recoil of valvular bioprosthesis after balloon deflation [65].

Infective endocarditis is a possible adverse event during follow-up. Melody TPV looks like it shows a higher incidence of infective endocarditis than Edwards Sapien THV [66]. Both Contegra conduit (Medtronic Inc., Minneapolis, MN, USA) and Melody TPV consist of a glutaraldehyde-preserved valve-containing bovine jugular vein graft. They are both characterized by similar incidence of infective endocarditis, suggesting an intrinsic mechanism related to their material and structure. Patel et al. suggested that the intrinsic biological features of Contegra conduits and their method of preparation may increase the thrombogenicity of grafts with consequent higher tropism for microorganisms [67]. Probably, a transvalvular residual gradient generates turbulence, which predisposes to thrombi formation, which exacerbates the turbulence, creating a vicious circle. The coupling of thrombi and blood-flow turbulence becomes a nidus for seeding by microorganisms. For these reasons, it is recommended to have life-long aspirin therapy, prophylaxis with antibiotics before surgical and/or oral invasive procedures, and to avoid significant transvalvular residual gradient. Malekzadeh-Milani et al. demonstrated a higher incidence of infective endocarditis after aspirin discontinuation and immediately after a right heart catheterization in patients with Melody TPV [68].

Table 6 summarizes the features and the indications of each transcatheter pulmonary valve.

## 9. Future Prospects

The biological valvular prostheses described are available to everyone in Europe with an approved CE mark. However, other prostheses are used in the world and are waiting to be available in Europe.

The Med-Zenith PT-Valve (Beijing Med-Zenith, Beijing, China) is a self-expandable valvular biological prosthesis designed to treat large native RVOT disfunction. The Med-Zenith PT-Valve is characterized by a nitinol wire frame covered with porcine pericardium and an inner three-leaflet porcine pericardial valve. Like the other self-expandable valves, a peri-procedural assessment by an angio CT scan is crucial to predict the procedural feasibility [69].

The Pulsta valve (TaeWoong Medical Co., Ltd., Gimpo-si, Gyeonggi-do, Republic of Korea) is a self-expandable valve with flared ends developed in South Korea to treat large native RVOT. The Pulsta valve is composed of a nitinol stent covered in porcine pericardium with an inner porcine pericardial valve. An RVOT landing zone < 29 mm is required to implant the prosthesis, and a balloon interrogation test of RVOT is often recommended [70].

The Meryl Myval THV (Meril Life Sciences Pvt. Ltd., Vapi, Gujarat, India) is a balloon-expandable valve characterized by a nickel–cobalt honeycomb framework with bovine pericardium leaflets and a proximal-end PTFE skirt to reduce the risks of paravalvular leakages. A balloon interrogation RVOT test is required to detect the landing zone. A landing zone < 31 mm is necessary to implant the valve, avoiding the risk of embolization [71].

Despite the scientific and technological progress of interventional cardiology that allowed the expansion of the patients susceptible to percutaneous treatment, in some unfavourable anatomies, the TPVR is not feasible. In some circumstances, a hybrid (surgical and percutaneous) allows for relief of the pulmonary disfunction by avoiding the cardiopulmonary bypass. The No-React Injectable Bio Pulmonic (Bio Integral Surgical Inc., Toronto, ON, Canada) can be implanted after sternotomy and surgical plication of the RVOT without the cardiopulmonary bypass. This hybrid approach reduces operation times and the adverse events related to cardiopulmonary bypass with improved outcomes compared to the conventional SPVR [72].

## 10. Conclusions

Transcatheter treatment of right heart disease in ACHD is a novel and charming conquest of interventional cardiology and biomedical engineering. The current marked valves have proven effective and safe, and they are the first choice of treatment when feasible. Recent research has improved the techniques of implantation of these devices. Novel systems and valvular bioprostheses have recently been developed to overcome these limits and to widen the range of percutaneous dealing. Further evidence is required to achieve a complete diffusion of these systems, even if the first results seem strongly promising, opening interesting perspectives about this field of congenital heart diseases in adults.

## Figures and Tables

**Table 1:** Proactive and conservative approach criteria. Patients with pulmonary regurgitation (RF ≥ 25%) and ≥2 of the following criteria.

	Proactive Approach	Conservative Approach
RVEDVi	>160 mL/m^2^	>180 mL/m^2^
RVESVi	>80 mL/m^2^	>95 mL/m^2^
RVEF	<47%	<40%
LVEF	<55%	<45%
QRS width	>160 ms	>180 ms

Abbreviations. LVEF: left ventricle ejection fraction, RF: regurgitant fraction, RVEDVi: right ventricle end-diastolic volume index, RVESVi: right ventricle end-systolic volume index, RVEF: right ventricle ejection fraction.

**Table 2:** Melody TPV: models and measurements.

Model	Bovine Jugular Vein	NuMed Platinum Iridium Stent	Length (Out of the Jar)	Landing Zone
Melody TPV 20	16 mm	CP-34 (8 crown-6 zig)	30 mm	16–20 mm
Melody TPV 22	18 mm	CP-34 (8 crown-6 zig)	28 mm	18–22 mm

**Table 3:** Edwards Sapien THV: models and measurements.

Model	Diameter	Length	Landing Zone
Edwards Sapien THV 20	20 mm	13.5 mm	16–19 mm
Edwards Sapien THV 23	23 mm	14.3 mm	18–22 mm
Edwards Sapien THV 26	26 mm	17.2 mm	21–25 mm
Edwards Sapien THV 29	29 mm	19.1 mm	24–27 mm

**Table 4:** Venus P-Valve: models and measurements.

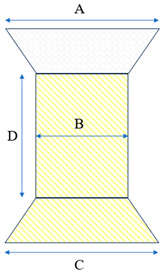	**Model**	**Specification**	**Diameter (mm)**			
			**A**	**B**	**C**	**D**
	L28P	P28-25	38	28	38	25
		P28-30				30
	L30P	P30-25	40	30	40	25
		P30-30				30
	L32P	P32-25	42	32	42	25
		P32-30				30
	L34P	P34-25	44	34	44	25
		P34-30				30
	L36P	P36-25	46	36	46	25
		P36-30				30

**Table 5:** Harmony TPV: models and measurements.

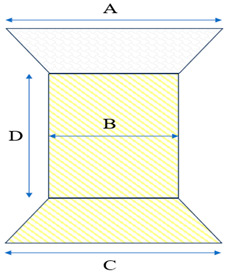	**Model**	**Diameter (mm)**			
		**A**	**B**	**C**	**D**
	Harmony TPV 22	32	22	41	55
	Harmony TPV 25	43	25	54	51

**Table 6:** Summary of Transcatheter pulmonary valve.

Model	Conduit RV-PA	Bioprosthetic PV	Native RVOT	Landing Zone
Melody TPV (balloon-expandable)	X	X		16–22 mm
Edwards Sapien THV (balloon-expandable)	X	X	X	16–27 mm
Venus P-Valve (self-expandable)			X	26–34 mm
Harmony TPV (self-expandable)			X	CT analysis
Alterra Adaptive Prestent (self-expandable)			X	27–38 mm

Abbreviations. PV: pulmonary valve, RV-PA: right ventricle—pulmonary artery, RVOT: right ventricle outflow tract. * Active endocarditis, clinical or biological signs of infection, and venous anatomy unsuitable to have the delivery sheath required are contraindications for transcatheter pulmonary valve replacement. ** The table summarizes the IFU features and indications of each pulmonary valve; however, off-label case reports in unconventional anatomy are reported for each valve.

## Data Availability

Not applicable.
